# A Bioinformatic Study on the Potential Anti-Vitiligo Activity of a *Carpobrotus edulis* Compound

**DOI:** 10.3390/molecules28227545

**Published:** 2023-11-11

**Authors:** Emna Trigui, Hanen Ben Hassen, Hatem Zaghden, Maher Trigui, Sami Achour

**Affiliations:** 1 Laboratory of Bioressources, Integrative Biology & Valorisation (BIOLIVAL), Higher Institute of Biotechnology of Monastir, Monastir University, Monastir 5000, Tunisia; triguiemna31@gmail.com (E.T.); trigui_m@yahoo.com (M.T.); samnaw2001@yahoo.fr (S.A.); 2 Laboratory of Probabilities and Statistics, Faculty of Sciences of Sfax, Sfax University, Sfax 3000, Tunisia; 3 Laboratory of Plant Molecular Physiology, Centre of Biotechnology of Borj-Cédria, Hammam-Lif 2050, Tunisia

**Keywords:** *Carpobrotus edulis*, vitiligo, drug design, pharmacophore, modeling, machine learning, 3D-QSAR, pharmacokinetic, modeling

## Abstract

The plant *Carpobrotus edulis* has traditionally been known for its wide applications in diseases, especially vitiligo, which is characterized by patches and white macules caused by the loss of melanocytes. One of the chemical treatments for vitiligo consists mainly of skin repigmentation and usually leads to a non-durable effect by inhibiting the Janus kinase (JAK) signal transduction (STAT pathway). JAK inhibitors generally block multiple JAK tyrosine kinases, which leads to secondary effects. In this study, natural molecules from *Carpobrotus edulis* were extracted and tested using a structure-based drug-design approach and pharmacophore modeling. The best-fit candidate from the extracted molecules was compared to the chemical molecules used. The results indicated a similarity between the chemical and natural ligands which suggested the potential use of the natural product against vitiligo. The main finding of this research work was the discovery of a new molecule extracted from a natural plant and the detection of its anti-vitiligo activity using an in-silico approach. This method can significantly reduce the cost of searching for potential medicinal molecules.

## 1. Introduction

Medicinal plants have been widely used in healthcare due to their abundance of secondary metabolites which are biologically active against toxicity and diseases [1]. Global studies have validated their efficiency, and some of the results lead to the development of plant-based medicines. A medicinal plant is a plant containing substances that can be dedicated to a therapeutic objective or that contain precursors for drug synthesis [2]. This definition requires the distinction to be made between medicinal plants that have scientifically proven therapeutic ingredients and plants that are considered medicinal but have not been scientifically studied. One such less-studied plant is *Carpobrotus edulis* (*C. edulis*), formerly known as *Mesembryanthemum*. *C. edulis* is a medicinal and edible succulent plant belonging to the order of *Caryophyllales* and the family of *Aizoaceae*. It is a common and diversified halophyte plant in South Africa; it is mainly abundant in the Eastern and Western Cape regions [3]. It is also abundant in North Africa (Algeria, Tunisia, and Libya). The plant grows rapidly, colonizes areas, and spreads along the soil, covering all surrounding surfaces and damaging other plant species. Due to its invasive nature, some European countries forbade its planting to protect other plant diversity [4]. So far, this plant seems to be known mainly for its ornamental use in decorations, soil stabilization, and erosion control.

*C. edulis* is reported to be a medicinal plant with high prominence and is traditionally used in many different disease treatments. Indeed, its juice and sap-filled leaves are habitually used to treat infections of the throat and mouth, digestive problems, dysentery, tuberculosis, and skin problems acquired from common infections. It has also been documented that *C. edulis* can be used against tooth and ear infections, to treat some oral or vaginal thrushes, and maladies such as diabetes and related issues [3,5]. It is also reported to be a candidate for future studies on novel and alternative therapies for the treatment of neurological disorders associated with low levels of acetylcholine in the brain [6]. Furthermore, *C. edulis* has been reported as being used as a traditional food additive that improves nutrient composition through food preservation [7], and its polysaccharides are valued for their application in the alimentary industry [8]. In addition, the qualitative phytochemical screening of *C. edulis* reveals twelve important phytochemicals. Phytochemicals are secondary metabolites associated with medicinal plant activities, habitually used for the treatment and control of life-threatening diseases. The screened extracts from different parts of plant are iridoids, saponins, carbohydrates/glycosides, coumarins, sulfates, flavonoids, anthraquinones, chlorides, alkaloids, cyanogenic glycosides, cardiac glycosides, unsaturated sterols/triterpenoids, and tannins. All these cited phytochemicals, except iridoids, exist in various parts of the *C. edulis* plant, mainly in the leaves, stem, and flowers. Indeed, the amount of phytochemicals varies depending on the part of the plant and the harvest season. It has been reported that leaves harvested during the autumn had higher amounts of these phytochemicals compared to other parts of the plant [9]. Furthermore, additional research has been carried out to study the bioactivity of *C. edulis*. Indeed, *C. edulis* extracts have an antimicrobial activity that has been studied by [9,10,11]. Another study of Omoruyi et al. [12] reported the antifungal activity of leaf substances extracted using different solvents such as hexane, acetone, ethanol, water, and essential oil. The extracts were evaluated on *C. edulis* pathogenic fungal strains: *Cryptococcus neoformans* and many species of *Candida* such as *C. krusei*, *C. rugosa*, *C. glabrata* and *C. albicans*. The essential oil extracts had the highest antifungal potency. Furthermore, the antioxidant activity of *C. edulis* was evaluated and was found to have a high antioxidant activity. The highest activity was observed in the stem and leaves [12,13,14].

In addition to the mentioned characteristics, the plant contents extracted with various solvents were found to possess anti-inflammatory properties as well. This was determined by their ability to inhibit the lipoxygenase enzyme, which is known to be involved in the inflammation process. A significant inhibitory potential was observed in this regard. In fact, the IC50 values revealed values lower than 100 µg/mL for the water and acetone extracts (IC50 values of approximately 60 ± 5 µg/mL and 22 ± 4, respectively). However, the methanol extracts demonstrated an IC50 of approximately 120 ± 17 µg/mL [15]. From all the cited properties, *C. edulis* seems to be an important medicinal plant used in ethnomedicine for the treatment of respiratory infections, toothache and earache, wounds, burns, hypertension, and other diseases, such as vitiligo. Actually, this plant is traditionally recognized for its use in vitiligo therapy; nevertheless, no scientific work has investigated this specific application yet.

Vitiligo is a depigmenting skin disorder caused by the loss of melanocytes, leading to the development of white macules and patches. Vitiligo is reported to be mediated by the targeted destruction of melanocytes by CD8^+^ T cells, with IFN-γ playing a central role in disease pathogenesis [16]. IFN-γ signaling uses the JAK-STAT pathway, and thus JAK inhibitors may be exploited to treat vitiligo [17,18]. In this case, and in most common cases, vitiligo treatment aims to achieve repigmentation of the damaged skin. Emerging evidence suggests that JAK inhibitors are efficient in alopecia areata, vitiligo, psoriasis, and atopic dermatitis. Further reports suggested that JAK inhibition may be broadly valuable for application in dermatology [19,20,21]. The research of small molecules as JAK inhibitors for vitiligo treatment is a promising area to be explored.

The main objective of this study is to set a scientific foundation for the use of *C. edulis* as a traditional medicinal plant for vitiligo therapy. This research work investigates the natural compounds from this plant as potential inhibitor ligands for the JAK complex; it is divided into the following two steps. The first step involves the extraction, using several solvents, of the different components of *C. edulis*, followed by the use of the GC-TOF-MS to characterize all compounds of the plant. The second step applies a bioinformatics approach to discern which potential ligands have the best affinity with the molecular target proteins involved in the vitiligo process. This step of the study focuses on structural comparisons between FDA-approved molecules and extracted *C. edulis* compounds.

## 2. Results and Discussion

### 2.1. Experimental Results

The solvents used in this work were water, ethanol, hexane, ethyl acetate and acetone. Water, ethanol and ethyl acetate have a lower health risk than other solvents such as hexane. In fact, traces of solvents may be found in the final products (plant extracts). For example, hexane has been shown to be neurotoxic to humans and has even been listed as a cause of occupational diseases in several European countries [22].

#### Characterization of the Components of *C. edulis*

The obtained extracts were analyzed by the GC-TOF-MS technique to identify the molecules obtained from the different parts of *C. edulis* in each of the solvents.

**Figure 1:**
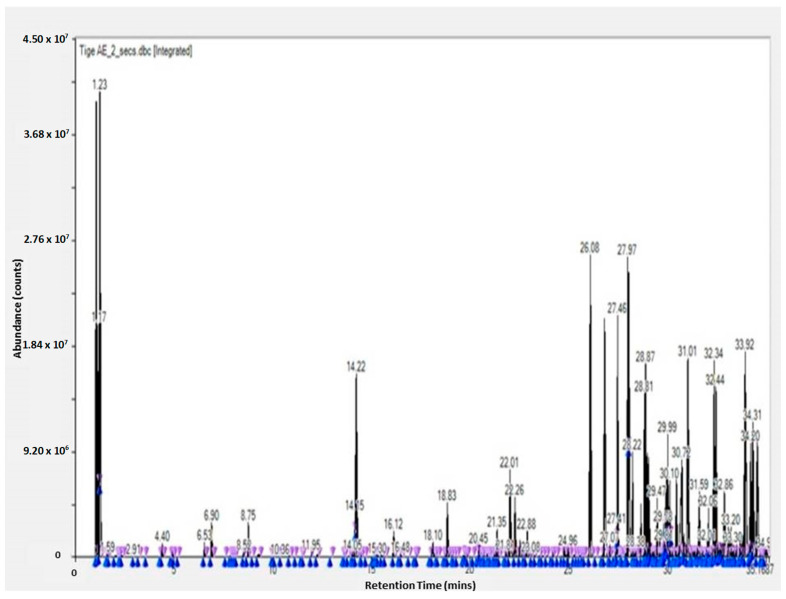
GC-TOF-MS results of the ethyl acetate extract from the stem of *Carpobrotus edulis*.

**Figure 2:**
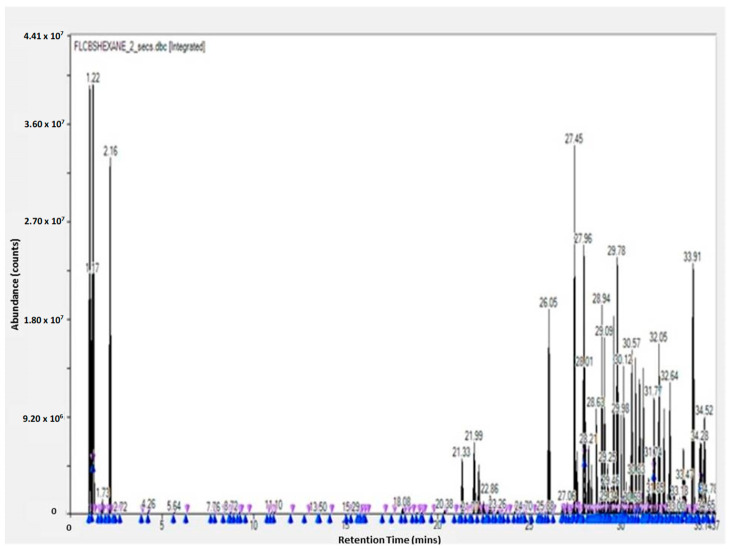
GC-TOF-MS results of the hexane extract of the flower of *Carpobrotus edulis*.

The GC-TOF-MS results of the obtained molecules include the following information: surface and percentage area, height, width, residence time, and molecular weight. This information is given for the leaves, flowers, and stem (see Supplementary Tables). These results are complementary to the work of other researchers [3,23]. The GC-TOF-MS results were obtained as chromatograms (Figure 1 and Figure 2), which revealed the residence time (min) relative to the abundance of the extract, respectively. Some of the obtained molecules are presented in Table 1 and Table 2, for stem ethyl acetate extract and for flower hexane extract, respectively.

All the information obtained from the GC-TOF-MS process was used to create a database of the *C. edulis* plant.

### 2.2. In Silico Study Results

- Creation of the *C. edulis* plant database

The 3D structure of each molecule of the *C. edulis* stem compound was obtained from the ChemBL database. All of the information on all of the structures was stored in a phase called the *C. edulis* phase of the Maestro11.2 software (Schrodinger). This information includes compound properties such as AlogP, HBA, HBD, and molecular weight.

- Identification of the molecular target

To treat vitiligo, the JAK 1/JAK 3 and JAK 1/JAK 2 molecular targets were, respectively, inhibited using JAK chemical inhibitors, tofacitinib and ruxolitinib. In our work, these two complexes were the targets of our review and study.

- Bioactivity of molecular target inhibitors

The inhibitors of the JAK 1/JAK 3 and JAK 1/JAK 2 complexes used in the current research were collected in July 2022. Their molecular information was extracted from the BindingDB database and supplemented to Maestro11.2 using the standard molecular property method. The information from the BindingDB database included 53 inhibitor ligands for the JAK 1/JAK 2 complex and 136 for the JAK 1/JAK 3 complex. In the BindingDB database, the ligand bioactivity of the molecular targets can vary significantly. For example, for the JAK 1/JAK 3 complex, the IC50 value of the molecule with the accession number BDBM50527406 was 2.6 nM. However, the bioactivity of the molecule with the accession number BDBM50530785 was 39,000 nM.

- Classification of molecular target inhibiting molecules

**Figure 3:**
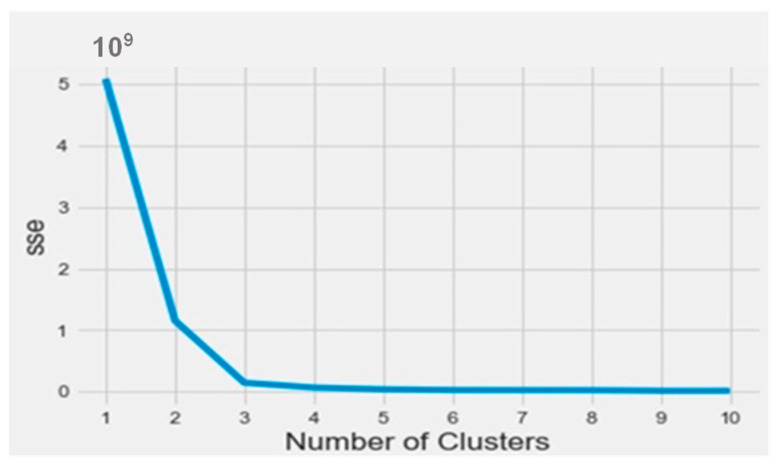
The SSE curve as a function of the number of clusters.

**Figure 4:**
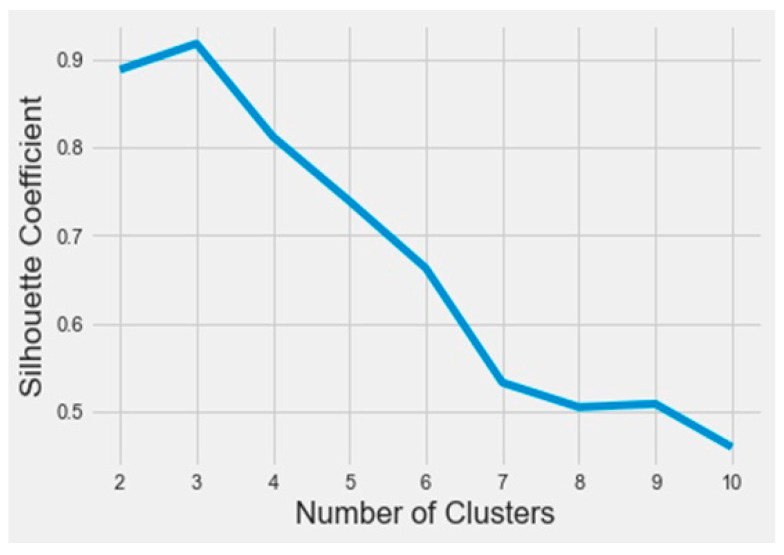
The silhouette curve as a function of the number of clusters.

To find out the bioactive molecule class, based on the lower IC50, a classification using the k-means method was implemented in Python on the Anaconda platform. The results were analyzed based on the parameters SSE (sum of squared errors) and silhouette (distance between the class centers). The obtained SSE value shown in Figure 3 indicates that the overall molecules had to be arranged into three different classes according to the elbow at class level three. Figure 4 represents the distance between the class centers, indicating that about 95% of all the data were already classified into three classes.

**Figure 5:**
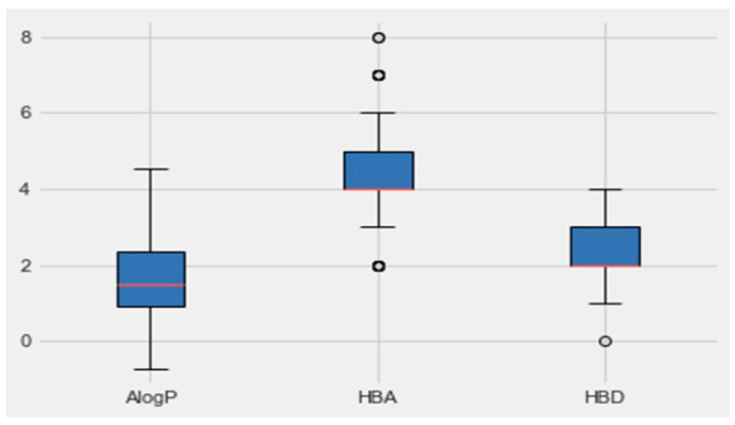
Boxplots of AlogP, HBA and HBD based on group activity. The red lines indicate the median values and the circles indicate the outliers.

**Figure 6:**
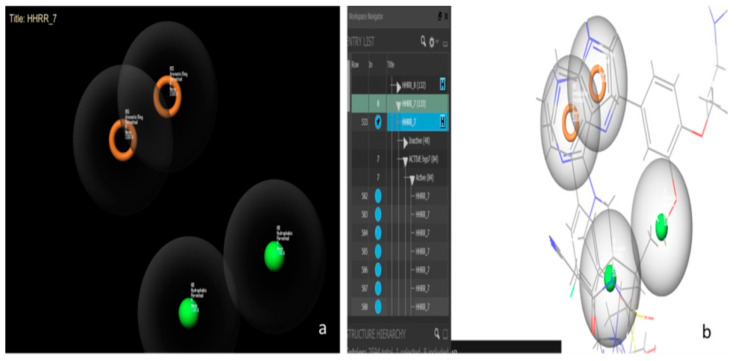
Hypothesis 7 created by Maestro11.2. (**a**) Represents the hydrophobic-hydrophobic-ring-ring functional group. (**b**) Represents some active molecules with respect to hypothesis 7.

An analysis of the properties of each class was performed by Python. Figure 5 represents the boxplot of each property of the active class (AlogP, the number of hydrogen-bond donors and acceptors HBD and, HBA, respectively). Based on the boundaries of the boxes in Figure 6, the results suggested that the most desirable regions for bioactivity were MW > 400, AlogP < 3, and HBA > 6. More details for all the other parameters, which are molecular weight, targets bioactivities, RO5 violations, rotatable bonds, QED weighted, CX BpKa, CX LogP, CX LogD, aromatic rings, inorganic flag, heavy atoms, HBA Lipinski, HBD Lipinski, RO5 violations (Lipinski), and molecular weight (monoisotopic), are presented in Table 3. The resulting quartiles of all the molecules of active classes were computed and used as a filter for all the molecules in the *C. edulis* database by the ligand preparation method (LigPrep).

- Creation of the pharmacophore model

Pharmacophores were created by Maestro11.2 based on the filter molecules of the active class. The results of pharmacophore creation are shown in Table 4. Twenty of the best hypotheses were selected, and the performance of each hypothesis is presented in Table 4. These hypotheses contain four and five features. The hypothesis with the highest AUC was chosen for the next step which is the screening of the *C. edulis* phase molecules.

Hypothesis 7 presents the best performance of all the hypotheses with an area under the curve (AUC) value of 0.98. Figure 6 presents this hypothesis, which was created by Maestro11.2 and formed by a hydrophobic-hydrophobic-ring-ring functional group (HHRR_7) (Figure 6a). There are 84 active molecules and 48 inactive molecules. All the active molecules have the same features and the same positions of hypothesis HHRR_7 (Figure 6b). 

**Figure 7:**
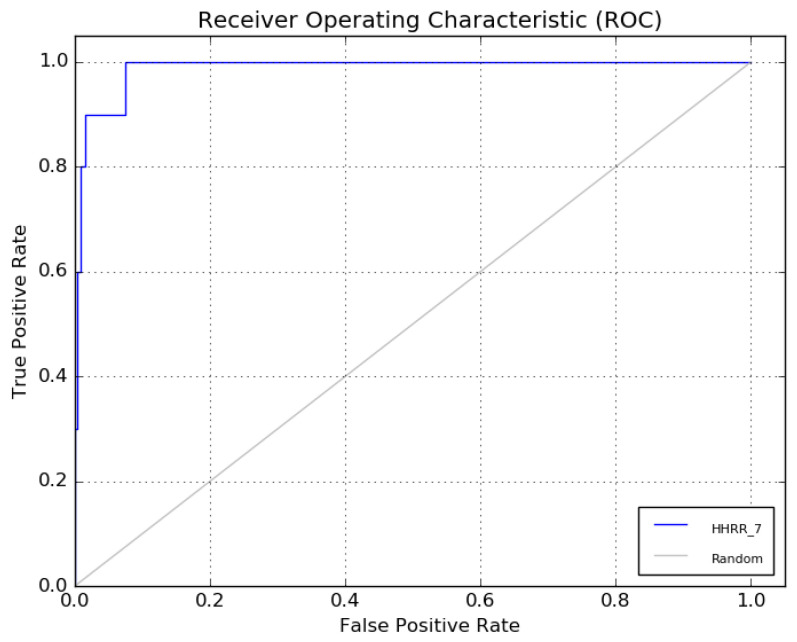
The ROC curve of hypothesis 7.

The receiver operating characteristic (ROC) curve of hypothesis 7 is presented in Figure 7.

- Filtering and Screening of the *C. edulis* phase

Table 5 represents the results of the screening of the pharmacophoric molecules of the stems and flowers compared to hypothesis 7. At the level of the flower, a single molecule (C_12_H_26_O_2_) with only one conformation was obtained which respects the pharmacophore; however, four molecules (C_16_H_17_N_3_O_2_, C_19_H_26_O_2_, C_20_H_30_O_2_, and C_19_H_23_NO_4_) respect hypothesis 7 in the stem. They have 23, 5, 1, and 1 conformations, respectively. These five molecules that were found were subjected to the next step of the pharmacokinetic analysis using the Swiss ADME program. 

**Figure 8:**
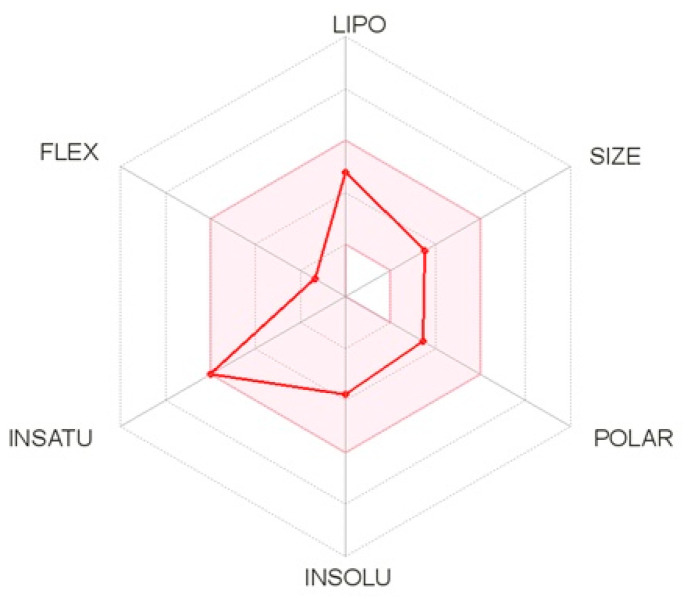
Bioavailability radar plot of the best molecule.

The pharmacokinetic properties of the five molecules were determined by the Swiss ADME method, which represents the properties in a six-axis landmark: LIPO (lipophilicity), FLEX (flexibility), SIZE, POLAR (polarity), INSATU (saturation) and INSOLU (solubility). From the bioavailability radar plot of the molecule C_16_H_17_N_3_O_2_ (Figure 8), which was extracted from the *C. edulis* stem, it can be observed that this molecule does not exceed the limits (pink area). Therefore, this molecule has acceptable properties of adsorption, distribution, metabolism, and excretion. This molecule was selected for the next molecular docking process.

- Molecular docking

**Figure 9:**
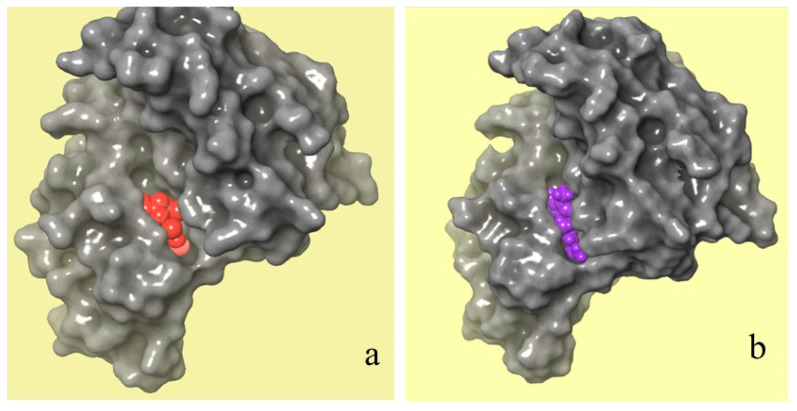
Crystal structures of JAK 1 and JAK 2 complexes with their chemical (**a**) and natural (**b**) inhibitors (red color refers to a chemical inhibitor, and purple color refers to C_16_H_17_N_3_O_2_ molecule extracted from *C. edulis* Stem).

Figure 9 shows a comparison between the molecular binding of natural and chemical ligands. The crystal structure of the JAK 1 and JAK 2 complexes with their inhibitors was extracted by the RCSB PDB database (Figure 9a), which shows the position of the chemical ligand (in red) in the JAK 1 and JAK 2 complexes. However, Figure 9b shows the position of selected natural ligands (in purple) in the JAK 1 and JAK 2 complexes. The obtained results of molecular docking show that the two molecules have the same attachment site in the JAK 1/JAK 2 inhibitor complex.

**Figure 10:**
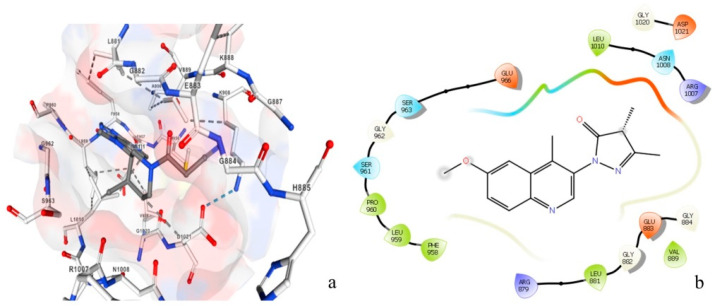
Amino acids attached to the JAK 1/JAK 2 complex inhibitor chemical molecule (**a**) versus amino acids attached to the natural molecule extracted from *Carpobrotus edulis* (**b**). Red refers to Oxygen, white refers to Carbon, blue refers to Nitrogen and pink refers to the hydrophobic zone.

Figure 10 represents the amino acids attached to the inhibitory chemical molecule of the JAK 1/JAK 2 complexes (Figure 10a) versus the amino acids attached to the natural molecule extracted by the plant *C. edulis* (Figure 10b). This figure clearly shows that the same amino acids are attached to the chemical and natural molecules (Leu1010, ASN1008, Ser963, Gly962, Leu881, Gly882, Glu883, and Val884,); therefore, they potentially have similar activity. These results confirmed that the chemical molecule can be replaced with the natural molecule extracted from the plant *C. edulis* which has anti-vitiligo activity.

- The 3D-QSAR machine learning prediction

A three-dimensional quantitative structure activity relationship (3D-QSAR) model was created using the fingerprint parameters as output variables and all the other molecular properties as inputs for the model. Four machine learning models (LR, SVM, RF, and NN) and a bagging/stacking process were used. The results of the learning tests are given by the R-squared values presented in Table 6. The best model was created using the bagging and stacking techniques. The R-squared coefficients were computed for each model. The best model was the one that was obtained by the bagging/stacking process with an R-squared value 0.999. This model will be validated using experimental data for IC50 and will be the subject of another study in the future. The stability of this natural potential molecule C_16_H_17_N_3_O_2_ will be studied for dynamic molecular analysis. 

## 3. Material and Methods

### 3.1. Experimental Study

- Biological material

This work was carried out on the different organs (leaves, stem and flowers) of the *C. edulis* plant collected from the natural land of the governorate of Monastir, Tunisia. 

The leaves were very thick and fleshy, waterlogged, and triangular in their cross- section. They were opposite and separated by internodes of several centimeters. They were 8 to 11 cm long. The stems were creeping or hanging (60 to 120 cm, up to 3 m), forming a kind of carpet on the ground that was up to several meters long. The flowers, terminal and solitary were yellow or pink, large in size (up to 12 cm in diameter), and formed by numerous yellow petals and stamens blooming in the sun toward the middle of the day.

The different organs were dried in an oven at a temperature of 50 °C for 72 h. After drying, these samples were ground to obtain a fine powder.

- Extraction using organic solvents

Compound extraction was performed using the Soxhlet technique. The used solvents were acetone at 75 °C, hexane at 69 °C, ethanol at 79 °C and ethyl acetate at 77 °C. A total of 50 g of each organ of the crushed plant was placed in a cartridge, introduced into a Soxhlet-type extractor, and equipped at its base with a 500 mL flask, into which 500 mL of solvent was introduced. The collected mixture was subjected to evaporation of the solvent on a rotary evaporator to remove all traces of the extraction solvents. This treatment was carried out at moderate temperature (40 °C). At the end, the concrete fraction was recovered.

- Identification of *C. edulis* compounds using derivatization

The derivatization reaction, which aims to increase the volatility of metabolites was initiated by adding 25 µL of pyridine and 50 µL of the silylating agent BSTFA (with 1% TMCS) to each sample, vortex mixed for 2 min, and tightly sealed and heated at 70 °C for 1 h. Under a gentle stream of nitrogen, the excess of the derivatization agent and the pyridine were removed. The dried residue was redissolved in 0.5 mL of n-hexane. Each sample was vortex mixed, after adding the internal standard (see Supplementary Information), and carefully transferred to GC autosampler vials for subsequent GC-TOF-MS analysis.

The identification of *C. edulis* compounds by the GC-TOF-MS™ instrument was set up under analytical conditions. GC analyses were achieved on an Agilent 7890B GC unit coupled to a Bench TOF-Select™ system (Markes International, Llantrisant, UK) including tandem EI equipped with a SepSolve preparation robot with an automated tool change mode lPAL3-RTC (Llantrisant, UK). Both hard and soft ionization at 70–12 eV were set for identity confirmation and exploring the complementarity of spectral and response. The front inlet and ion source temperatures were both kept at 250 °C. The MS optimization option was set to operate in Tandem Ionisation™ with a full scan mode with a mass range of *m*/*z* 45–1000 m and an acquisition frequency of 50 Hz; the filament voltage was set at 1.50 V. The column used was a DB-1 GC column (Agilent Technologies, 5301 Stevens Creek Blvd, Santa Clara, CA 95051, USA) with a length of 20 m, an internal diameter of 0.18 mm, and a film thickness of 0.18 µm. Carrier gas used was helium at a flow rate of 1 mL/min. Split ratio for the injector was set to 1:10, with a total injection volume of 1 µL. The oven temperature program ranged from 70 °C (2 min) to 120 °C at 10 °C min^−1^ and, then to 320 °C (1 min) at 4 °C min^−1^.

For whole-grain flour samples, 2.0 μL of the derivatized solution was analyzed under the following conditions: split/splitless injector in a split mode, split ratio 1:20, and an injector temperature of 300 °C.

### 3.2. In Silico Study for Carpobrotus Compounds

- Molecular target

In recent years, multiple JAK inhibitors have demonstrated their efficacy in diseases such as rheumatoid arthritis, myelofibrosis, and polycythemia vera. The review work of Relke and Gooderham [24] indicated that the JAK 1/JAK 2 and JAK 1/JAK 3 complexes are the molecular targets involved in the process of vitiligo disease. Ferreira et al. [25] worked on the ligands tofacitinib for the JAK 1/JAK 3 complex and they worked on the ligand ruxolitinib for the JAK 1/JAK 2 complex. The target of the current study is the JAK 1/JAK 2 complex.

- Creation of a phase for the *C. edulis* plant

A phase, which is a database in the Maestro11.2 software, was created. All the GC-TOF-MS extracted molecules from the plant *C. edulis* are stored in this phase, and all the physicochemical properties of each molecule such as the octanol water partition coefficient (AlogP), the number of hydrogen bond donors (HBA), the number of hydrogen bond acceptors (HBD), and the molecular weight (MW) are included. Additionally, violations of the Lipinski rules [26] of each molecule were integrated and computed. These properties are essential for the next screening step.

- Data preparation and filtration

BindingDB is a public database accessible online that integrates information on the bioactivity of the given molecules. BindingDB was searched for known JAK 1/JAK 2 and JAK 1/JAK 3 protein complex inhibitors. The search identified 53 compounds for the JAK 1/JAK 2 inhibitors and 136 compounds for the JAK 1/JAK 3 inhibitors (September 2022). In the BindingDB database, the bioactivity (IC50) of the ligands of the molecular targets varied from 2.6 to 30,000 nM. The k-means method was carried out to classify all the ligands according to their IC50 bioactivity. This procedure was implemented in Python on the Anaconda platform. The analysis of this ranking is based on the sum of the square of errors (SSE) and silhouette (distance between class centers). The analysis of the properties of each class was performed. The best bioactive class with a low value of IC50 was used in the next step of analysis which was the filtration of all the molecules.

- Creation of a pharmacophore model

Hypothetical pharmacophore models were developed based on the common functional group of the most bioactive molecules. To determine the best hypothesis that respects the pharmacophore model, the receiver operating characteristic curve (ROC) curve was drawn. Graphically, this curve gives the rates of the true- and false-positive pharmacophore models. The models with an ROC higher than 0.8 were accepted and used for the next step.

- Filtering and screening of the *C. edulis* phase

All the *C. edulis* molecules obtained from the experimental data are stocked in a database. This step screens for molecules in the database. The screened molecules must respect the characterization of the best bioactive class of molecules and the selected pharmacophore model. In addition, the accepted molecules from the screening step were tested by the SwissADME website. This open-source platform enables the prediction of pharmacokinetic properties (ADME parameters: adsorption, distribution, metabolic, extrusion) [27].

- Molecular docking

The molecules retained in the screening step were used in the molecular docking process, which provided information on the position of the ligands in the protein. A comparison should be made between the position of the crystal ligands and the position of the natural ligands. The binding affinity (Kcal/mol) of the ligand to the receptor and the RMSD (root mean square difference) parameter were used to classify the different conformations of all these ligands. The ligands with a lower binding energy and RMSD were selected and considered potential inhibitors of the molecular target. The selected molecules were used for the next step.

- Three-dimensional quantitative structure–activity relationship (3D-QSAR) machine learning prediction

The bioactivity of the selected *C. edulis* molecules from the docking process was predicted using a 3D-QSAR machine learning model.

Machine learning models are used to make 3D-QSAR predictions. The results provided by the different models are combined to obtain the majority prediction. This technique provides better performance than when the models are used separately. This is due to the law of large numbers; indeed, the more models we have, the better the collective performance. The models used must meet two major criteria: the first is that the crowd must meet at least 50% performance and that the crowd has a minimum of diversity. To reach this goal, different models have been used; multiple linear regression (MLR), support vector classifiers (SVCs), neural networks (NNs) and random forests (RFs). A new model that outperformed the best models was developed using bagging and stacking techniques [28,29]. Bagging consists of creating multiple copies of the same model, and training each copy on a random part of the dataset. For that, a bootstrapping technique is used. This consists of replacing the data near each draw that have been selected in the dataset. Stacking consists of training a machine learning model on top of the predictions of the crowd, i.e., instead of gathering the results of the models to obtain a majority prediction, a final estimator is asked to learn and predict the final result based on the previous predictions. The algorithm used for this technique is the VotingRegressor. The results of each model are aggregated to make the final 3D-QSAR predictions.

## 4. Conclusions

In this paper, bioactive molecules from the *C. edulis* plant responsible for anti-vitiligo activity were extracted, analyzed, and compared to chemical molecules used in anti-vitiligo treatment. Indeed, for the extracts analyzed with GC-TOF-MS, an in slico study was applied to detect potential anti-vitiligo molecules. This study revealed the existence of a natural molecule C_16_H_17_N_3_O_2_, extracted from the *C. edulis* stem using an ethyl acetate solvent, comparable to the chemical one used for the treatment of vitiligo. This natural molecule respects the ADME parameters, which confirm and explain the traditional use of the *C. edulis* plant against vitiligo, and which may suggest that the chemical treatment be replaced with the natural treatment.

## Figures and Tables

**Table 1:** Phytomolecules identified in ethyl acetate extract from the stem of *Carpobrotus edulis*.

Residence Time (min)	Compounds	Formula
3.5	Pentitol, 1,3-didesoxy-tris-*O*-(trimethylsilyl)-	C_14_H_36_O_3_Si_3_
7.9	Decanal, *O*-methyloxime	C_11_H_23_NO
8.9	Bisphenol A monomethyl ether, TMS derivative	C_19_H_26_O_2_Si
10.8	Ethanol, 2-(trimethylsilyl)-	C_5_H_14_OSi
14.4	6-Ethyl-5,6-dihydro-2*H*-pyran-2-one	C_7_H_10_O_2_
20.5	7-Isoquinolinol, 1,2,3,4-tetrahydro-1-[(3-hydroxy-4-methoxyphenyl)methyl]-6-methoxy-2-methyl-, (*S*)-	C_19_H_23_NO_4_
26.3	4-Oxazolecarboxylic acid, 4,5-dihydro-2-phenyl-, 1-methylethyl ester	C_13_H_15_NO_3_
28.6	1-Decanol, 2-hexyl-	C_16_H_34_O
30	Hexadecanoic acid, 4-[(trimethylsilyl)oxy]butyl ester	C_23_H_48_O_3_Si
32	A-Norcholestan-2-one, (5α)-	C_26_H_44_O
35	10,12-Tricosadiynoic acid, TMS derivative	C_26_H_46_O_2_Si

**Table 2:** Phytomolecules identified in the hexane extract of *Carpobrotus edulis* flower.

Residence Time (min)	Compound	Formula
2.4	cis-2-Decenoic acid	C_10_H_18_O_2_
4.257	hexanoic acid	C_6_H_12_O_2_
9.2	Bisphenol A monomethyl ether, TMS derivative	C_19_H_26_O_2_Si
14.17	S-Methyl-L-cysteine	C_4_H_9_NO_2_S
15.29	3-Octenoic acid, TMS derivative	C_11_H_22_O_2_Si
18.865	1,8-cis-Undecadien-5-yne 3,7-bis-trimethylsilyl ether	C_17_H_32_O_2_Si_2_
21.987	Neophytadiene	C_20_H_38_
23.64	Dioctyl phthalate	C_24_H_38_O_4_
28.74	2-Amino-2-methyl-1,3-propanediol	C_4_H_11_NO_2_
30.625	Sulfurous acid, dodecyl 2-propyl ester	C_15_H_32_O_3_S
31.4	1-Pentacosanol, TMS derivative	C_28_H_60_OSi
34.78	Hexacosane	C_26_H_54_

**Table 3:** The resulting quartiles of all molecules of active classes.

Quartie	MW	T	B	RO5V	RB	QEDW	CX BpKa	CX LogP	CX LogD	AR	IF	HA	HBAL	HBDL
25%	372.45	6.00	7.00	0.00	4.00	0.54	0.73	0.71	0.27	2.00	1.00	26.00	7.00	2.00
75%	428.56	25.00	5.00	0.00	5.00	0.73	10.05	2.72	1.86	3.00	1.00	31.00	9.00	5.00

Legend: MW: molecular weight, T: targets, B: bioactivities, ROV: RO5 violations, RB: rotatable bonds, QEDW: QED weighted, AR: aromatic rings, IF: inorganic flag, HA: heavy atoms, HBAL: HBA Lipinski, and HBDL: HBD Lipinski.

**Table 4:** The 20 best hypotheses created by Maestro11.2.

Hypothesis	Phase Hypo Score	EF1%	BEDROC160.9	ROC	AUAC	Ave. Outranking Decoys	Total Actives	Matches	Excluded Volumes
HHRR_8	1.27	50.50	0.71	0.98	0.98	16.70	10	4 of 4	No
HHRR_7	1.27	60.60	0.69	0.99	0.98	11.80	10	4 of 4	No
HHRR_6	1.27	70.70	0.85	0.89	0.93	8.44	10	4 of 4	No
HHRR_5	1.26	50.50	0.66	0.97	0.97	28.10	10	4 of 4	No
HHRR_4	1.29	60.60	0.76	0.98	0.98	15.10	10	4 of 4	No
HHRR_3	1.28	50.50	0.73	0.97	0.97	27.90	10	4 of 4	No
HHRR_2	1.26	40.40	0.61	0.97	0.97	25.10	10	4 of 4	No
HHRR_1	1.25	50.50	0.69	0.87	0.91	28.78	10	4 of 4	No
HHHR_2	1.14	40.40	0.56	0.60	0.77	6.83	10	4 of 4	No
HHHR_1	1.14	40.40	0.58	0.59	0.77	14.83	10	4 of 4	No
HHHRR_7	1.18	50.50	0.62	0.50	0.74	1.00	10	5 of 5	No
HHHRR_6	1.16	40.40	0.56	0.50	0.74	2.60	10	5 of 5	No
HHHRR_5	1.18	40.40	0.59	0.50	0.74	2.40	10	5 of 5	No
HHHRR_4	1.16	30.30	0.53	0.50	0.74	3.40	10	5 of 5	No
HHHRR_3	1.18	40.40	0.60	0.50	0.74	4.00	10	5 of 5	No
HHHRR_2	1.19	40.40	0.61	0.50	0.74	2.20	10	5 of 5	No
HHHRR_1	1.19	50.50	0.66	0.50	0.74	0.40	10	5 of 5	No
AHHRR_3	1.24	40.40	0.56	0.79	0.88	14.62	10	5 of 5	No
AHHRR_2	1.23	40.40	0.56	0.79	0.88	15.25	10	5 of 5	No
AHHRR_1	1.24	40.40	0.63	0.79	0.88	13.12	10	5 of 5	No

**Table 5:** Screening results of the pharmacophoric molecules of the stem and flower compared to hypothesis 7.

Organs	Solvents	Hypothesis	Molecules	Conformations
Flower	Hexane	(HHRR_7)	C_12_H_26_O_2_	1
Stem	ethyl acetate	(HHRR_7)	C_16_H_17_N_3_O_2_	23
Stem	ethyl acetate	(HHRR_7)	C_19_H_26_O_2_	5
Stem	ethyl acetate	(HHRR_7)	C_20_H_30_O_2_	1
Stem	ethyl acetate	(HHRR_7)	C_19_H_23_NO_4_	1

**Table 6:** R-squared test results for all the trained models.

Model	R-Squared Test
LR	0.880
SVC	0.680
RF	0.942
NN	0.998
Bagging/stacking	0.999

## Data Availability

The data presented in this study are available in supplementary material.

## Supplementary Materials

The following supporting information can be downloaded at: https://www.mdpi.com/article/10.3390/molecules28227545/s1, Figure S1. Data analysis workflow, Table S1: The Mixed Standards (MS) used in the GC-TOF-MS procedure, Table S2. Performance Comparison of the One-Phase vs Two-Phase Alignment with the CC Samples, Table S3: Metabolites extracted from leaves, stem and flowers of *C. edulis*.
