# Supplementary material for: A Bioinformatic Study on the Potential Anti-Vitiligo Activity of a Carpobrotus edulis Compound
Source: Molecules. 2023 Nov 11;28(22):7545. doi: 10.3390/molecules28227545 (PMC10673461; doi:10.3390/molecules28227545)
Supplement: Supplementary file 1 [file molecules-28-07545-s001.zip › Figure S1.pdf]

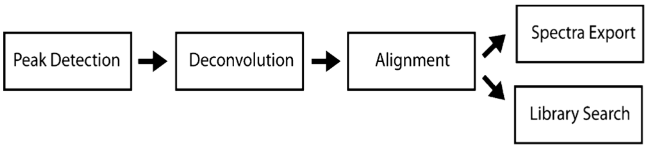

Figure S1. Data analysis workflow.

All the standard mixtures and serum samples were prepared, derivatized, and analyzed. In brief, after TMS derivatization, each 1 μL aliquot of the derivatized solution was injected in splitless mode into Agilent 7890B GC unit coupled to a Bench TOF-Select™ system (Markes International, Llantrisant, UK). Derivatization reaction aimed to increase the volatility of metabolites was then initiated by adding 25 μL of pyridine and 50 μL of the silylating agent BSTFA (with 1% and heated at 70°C for 1 h. Under a gentle stream of nitrogen, the excess of the derivatization agent and the pyridine were removed. The dried residue was re-dissolved in 0.5 mL of n-hexane. Each sample was vortex-mixed, after adding internal standard, and carefully transferred to the GC autosampler vials for subsequent GC-TOF-MS analysis.
