# Supplementary material for: A Bioinformatic Study on the Potential Anti-Vitiligo Activity of a Carpobrotus edulis Compound
Source: Molecules. 2023 Nov 11;28(22):7545. doi: 10.3390/molecules28227545 (PMC10673461; doi:10.3390/molecules28227545)
Supplement: Supplementary file 1 [file molecules-28-07545-s001.zip › Table S1.pdf]

Table S1: The Mixed Standards (MS) used in the GC-TOF-MS procedure

No	Compounds	ET(min)	Sample injection (ng)	NIST Score
1	L-!-alanine	6.2908	70	949
2	L-leucine	8.51	105	896
3	L-Proline	8.8917	94	762
4	Glycine	9.0025	141	957
5	Succinic acid	9.13	94	924
6	L-Serine	9.721	94	772
7	pipecolinic acid	9.815	141	886
8	beta-Alanine	10.645	188	953
9	4-Hydroxy-L-proline	11.8733	94	700
10	trans-Cinnamic acid	12.2384	188	866
11	L-cysteine	12.248	94	0
12	creatinine， anhydrous	12.248	141	937
13	!-Ketoglutaric acid	12.5142	188	933
14	L-asparate	12.605	188	954
15	L-Phenylalanine	13.1708	141	604
16	n-Dodecanoic acid	13.5467	141	864
17	L-(+)-Arabionse	13.5758	141	736
18	DL-Homocysteine	13.6375	281	822
19	L-Asparagine	13.457	188	797
20*	L-(+)-Rhamnose monohydrate	14.3375	141	910
		14.4433		876
21	L-(-)-arabitol	14.3833	234	952
22	1,4-diaminobutane	14.675	117	938
23	L－Ornithine monohydrochloride	15.8158	234	718
24	1,5-Diaminopentane dihydrochloride/Cadaverine	16.13	281	947
25	n-Tetradecanoic acid(myristic acid)	16.3825	141	548

26*	D-Fructose	16.6117	141	940
		16.7575		943
27	L-Histidine	17.3875	281	766
28	Indol-3-acetic acid	18.1075	188	925
29	Palmitic acid/hexadecanoic acid	19.5183	234	938
30	Dopamine hydrochloride	19.9408	234	946
31	3-indolepropionic acid	20.0175	117	938
32	Oleic acid	22.2967	281	946
33	n-Octadecanoic acid	22.7617	469	956
34	uridine	25.36	375	803
35	n-Eicosanoic acid	25.3725	703	912
36	Sucrose	26.7083	234	950
37	estradiol	27.1392	375	955
38*	Testosterone	27.3892,	938	967
		27.4458		973
Average				855.097561

Mixed Standards (MS) Samples: 38 standard compounds were carefully selected and mixed with known ratios. Criteria for selecting those compounds are the following. (i) They should contain different classes of compounds that include amino acids, retention times of compounds are spaced across the entire 30-minute time range.
