# Supplementary material for: A Bioinformatic Study on the Potential Anti-Vitiligo Activity of a Carpobrotus edulis Compound
Source: Molecules. 2023 Nov 11;28(22):7545. doi: 10.3390/molecules28227545 (PMC10673461; doi:10.3390/molecules28227545)
Supplement: Supplementary file 1 [file molecules-28-07545-s001.zip › Table S2.pdf]

Table S2. Performance Comparison of the One-Phase vs Two-Phase Alignment with the Calibration Curve Samples

RT (min)	compound name	One-phase alignment		Two-phase alignment	
		SmpNr	Score	SmpNr	Score
11.065	Decanoic acid	11	887	11	893
13.443	Laurate	11	855	11	863
14.559	Tridecanoic acid	11	883	11	890
16.626	Pentadecanoic acid	11	690	11	836
17.481	Palmitoleic acid	11	NA	11	880
17.74	Palmitic acid	3	605	11	819
18.852	Heptadecanoic acid	2	744	11	876
19.691	Linolic acid	10	479	11	798
19.768	Linolenic acid	6	NA	7	676
19.799	Oleic acid	Nab	NA	11	494
19.848	Elaidic acid	3	769	3	795
20.076	Stearic acid	11	749	11	914
20.464	8.11.14-Eicosatrienoic acid	NA	NA	11	840
21.254	Arachidonic acid	9	750	11	929
21.298	Eicosapentaenoic acid	2	651	11	812
21.567	11.14-Eicosadienoic acid	11	855	11	882
21.601	11-Eicosenoic acid	10	717	11	880
21.773	Arachidic acid	6	NA	11	NA
22.504	Decosahexaenoic acid	11	813	11	837
Average		8	746	10	829

Ten calibration curve samples were prepared at different dilutions from the original mixture of 19 fatty acid standards. These fatty acids vary in biochemical properties with different numbers of carbons and double bonds, or different double bond positions. A SmpNr refers to the total number of samples in which a compound was observed. b NA means that the corresponding metabolite was not detected with high confidence because the matching score is below the cutoff value that was set at 750.
